# Safe Integration of Physician Assistants in German Ambulatory Care: A Narrative Review and Practical Supervision Framework

**DOI:** 10.7759/cureus.112563

**Published:** 2026-07-13

**Authors:** Stefan Bigge

**Affiliations:** 1 Dermatology, Praxis Bigge Ungerechts, Cologne, DEU

**Keywords:** ambulatory care, delegation, germany, interprofessional care, patient safety, physician assistant, physician associate, primary care, role clarity, supervision

## Abstract

Physician Assistants (PAs) are increasingly discussed as a potential component of ambulatory care teams in Germany, particularly in response to physician workforce shortages, rising clinical and administrative workloads, and the need for sustainable team-based care models. However, international experience suggests that the integration of PAs should not be viewed primarily as a simple workforce expansion strategy, but rather as a matter of patient safety, role clarity, supervision, and governance. Ambiguous professional titles, unclear delegation boundaries, inadequate physician oversight, and the use of PAs as substitutes for physicians in undifferentiated clinical encounters may create avoidable safety risks and undermine trust among patients and healthcare professionals. This narrative review summarizes relevant international evidence, recent safety-oriented regulatory developments, and the specific legal and organizational context of German ambulatory care. It also proposes a practical supervision framework for the safe integration of PAs into general practices, specialist practices, professional practice partnerships, and medical care centers. The proposed framework emphasizes six key domains: transparent role identification, clearly defined delegation boundaries, tiered supervision, structured escalation criteria, documentation standards, and continuous quality monitoring. PAs may contribute meaningfully to ambulatory care when integrated as supportive members of physician-led teams. Safe implementation requires that delegated practice remains clearly distinct from autonomous substitution, that physicians retain overall clinical responsibility, and that patients clearly understand the professional role of the individual providing their care.

## Introduction and background

Previous work has discussed the general opportunities and challenges associated with Physician Assistants (PAs) in German outpatient care [1]. Building on that broader perspective, the present article adopts a narrower, safety-oriented focus by examining the supervision, delegation, and governance structures required for the safe integration of PAs into German ambulatory care.

Ambulatory care systems in many high-income countries face increasing pressure from demographic change, the growing burden of chronic disease, rising patient expectations, limited appointment capacity, and increasing administrative workload. Germany is no exception. The German Medical Association reported that, as of December 31, 2025, 43,444 professionally active physicians were older than 65 years, and a further 60,826 were between 60 and 65 years of age; overall, 23.4% of the professionally active medical workforce was aged 60 years or older [2]. In statutory ambulatory care, the Federal Association of Statutory Health Insurance Physicians reported that the number of contracted physicians and psychotherapists increased in 2025, but the available full-time clinical capacity did not increase proportionally because of part-time employment and changing work patterns [3]. Although these developments do not, in themselves, justify replacing physicians, they help explain why team-based care and structured delegation are receiving increasing attention.

In Germany, a PA is an academically trained health professional who supports physicians by performing delegable medical tasks within a physician-led team. Unlike physicians, PAs are not licensed to practice medicine independently and do not complete postgraduate medical specialty training. Their role is therefore not intended to provide a parallel pathway to independent medical practice.

For the purposes of this manuscript, ambulatory care is used as an umbrella term for physician-led outpatient medical care provided outside of inpatient hospital admission. It encompasses general practices, specialist practices, professional practice partnerships, and medical care centers, and should not be equated with primary care alone. Primary care first-contact encounters often involve undifferentiated symptoms and substantial diagnostic uncertainty. By contrast, specialist outpatient follow-up, post-procedural review, and the management of stable chronic diseases generally occur within more narrowly defined clinical contexts and may be more suitable for protocol-based PA support. This distinction is fundamental because the safety requirements for assessing a stable postoperative wound differ considerably from those for evaluating a new presentation with chest pain, dyspnea, syncope, abdominal pain, headache, or neurological symptoms.

In Germany, the legal and contractual framework governing delegation in statutory ambulatory medical care is based not on the autonomous substitution of physicians but on the delegation of defined medical tasks to qualified non-physician personnel under physician responsibility. The Federal Association of Statutory Health Insurance Physicians identifies Annex 24 of the Federal Master Agreement for Physicians as the governing agreement for the delegation of medical services in statutory ambulatory care, including a non-exhaustive catalogue of delegable tasks and the corresponding requirements for delegation [4]. Similarly, the 2025 position paper of the German Medical Association defines Physician Assistance as a supportive, academically trained profession that performs delegable medical activities, provided that the task is not legally reserved for personal performance by a physician and that statutory physician-only responsibilities are respected [5].

Recent German reforms also reflect a broader movement toward competence-based redistribution of responsibilities among non-physician health professionals. The Act on the Expansion of Competencies and Reduction of Bureaucracy in Nursing entered into force on January 1, 2026 [6]. This legislation primarily concerns the nursing profession and aims to expand the responsibilities of nursing professionals, including the more independent performance of selected healthcare tasks. It should not, however, be interpreted as a direct regulatory framework for PAs. Rather, it illustrates an important structural distinction: whereas recent nursing reforms increasingly emphasize expanded competencies and, in selected areas, more independent practice, the German PA model remains firmly anchored in physician-led delegation. For PAs, this context reinforces, rather than diminishes, the need for a clearly defined framework for supervision and delegation.

Internationally, the PA role has evolved differently across jurisdictions. In the United States, PAs have been established for several decades. In the United Kingdom, the term PA became more widely adopted, although recent policy discussions have again emphasized the importance of clear patient-facing professional titles. In the Netherlands and other healthcare systems, comparable roles have been introduced as part of broader workforce and skill-mix strategies. However, training duration, regulatory frameworks, prescribing authority, supervision requirements, professional identity, and public understanding vary substantially across countries. Consequently, evidence and policy lessons from one jurisdiction cannot be transferred directly to Germany without careful consideration of legal responsibility, physician availability, reimbursement structures, and the complexity of ambulatory clinical presentations.

The central question, therefore, is not whether PAs can contribute to German ambulatory care, but under which conditions they can be integrated safely. This narrative review argues that PAs should not be viewed as autonomous substitutes for physicians, particularly during undifferentiated first-contact presentations. Instead, their integration should be grounded in supervised delegation, transparent communication of professional roles, clearly defined escalation pathways, and continuous quality monitoring.

## Review

Methods of literature selection

This article was designed as a targeted narrative review with an evidence-informed practical framework. It was not intended to be a systematic review, meta-analysis, or PRISMA-guided scoping review. No statistical synthesis was performed because the objective was to integrate heterogeneous clinical, implementation, regulatory, and policy literature into a practical supervision framework for German ambulatory care.

A PubMed/MEDLINE search was conducted on June 18, 2026. The primary search period extended from January 1, 2011, to June 18, 2026, as the most relevant literature on PA implementation in the United Kingdom and Germany emerged during this timeframe. Earlier publications were included selectively when they provided foundational evidence on PAs in primary care, patient safety, or structured communication. Publications in English and German were considered. Articles were eligible for inclusion if they addressed PAs or Physician Associates in relation to primary care, ambulatory care, outpatient care, team-based care, supervision, role clarity, patient safety, quality of care, cost-effectiveness, task delegation, or implementation in Germany. Articles were excluded if they were unrelated to clinical or health system implementation, focused exclusively on non-comparable settings without transferable lessons, or consisted primarily of opinion pieces that were not relevant to the proposed framework. Official policy documents were identified through government, professional, and regulatory websites in Germany and the United Kingdom, including those of the Federal Association of Statutory Health Insurance Physicians, the German Medical Association, the Federal Ministry of Health, the Department of Health and Social Care, the General Medical Council, and NHS England.

The PubMed search used combinations of the search terms presented in Table 1. The search was supplemented by backward citation tracking of key systematic reviews and targeted retrieval of relevant German regulatory and professional documents. Approximately 120 PubMed records and official documents were screened at the title, abstract, or full-text level for relevance to PA implementation, supervision, delegation, patient safety, and transferability to German ambulatory care. Forty-two unique sources were ultimately selected for citation in the revised manuscript, comprising 26 scholarly publications related to PAs, Physician Associates, or closely related advanced-practice workforce models; 5 German regulatory, workforce, or policy documents; 3 UK regulatory or policy documents; 2 German sources on task delegation involving other non-physician roles; 2 reviews addressing task shifting or nurse substitution; and 4 sources on diagnostic safety, interprofessional collaboration, structured communication, or clinical handover. As this was a narrative review, no formal risk-of-bias assessment was performed. Instead, the synthesis explicitly distinguishes direct evidence on PAs from indirect conceptual evidence and highlights the limitations of transferring findings across different countries and healthcare settings.

**Table 1:** Narrative literature search and source-selection approach Source: Author-reported narrative review method and source-selection process. This table is intended to improve transparency and traceability; it does not convert the manuscript into a systematic review.

Element	Description used in this narrative review
Search date	PubMed/MEDLINE searched on June 18, 2026; official policy sources accessed primarily on June 18, 2026
Date range	Main search period: January 1, 2011, to June 18, 2026; older sources included selectively when foundational for PA primary care, patient safety, or structured communication
Languages	English and German publications or official documents were included
Core PubMed concepts	"physician assistant" OR "physician associate" combined with "primary care", "ambulatory care", "outpatient care", "Germany", "supervision", "delegation", "role clarity", "patient safety", "quality of care", "health workforce", "cost-effectiveness"
Example PubMed search strings	("physician assistant" OR "physician associate") AND ("primary care" OR "general practice" OR "ambulatory care" OR "outpatient care"); ("physician assistant" OR "physician associate") AND (supervision OR delegation OR "role clarity" OR "patient safety"); ("physician assistant" OR "physician associate") AND Germany; ("task shifting" OR delegation OR substitution) AND "primary care"
Policy-source identification	Targeted retrieval from official German and UK websites: KBV, Bundesärztekammer, Bundesministerium für Gesundheit, Department of Health and Social Care, General Medical Council, and NHS England
Inclusion focus	Direct PA/Physician Associate evidence; German PA literature; primary-care and ambulatory-care implementation studies; supervision and patient-safety literature; official policy or regulatory documents relevant to Germany or the UK
Exclusion focus	Sources without relevance to PA implementation, supervision, delegation, patient safety, or transferability to German ambulatory care; non-clinical opinion pieces without framework relevance
Approximate screening and final selection	Approximately 120 records or documents were screened; 42 sources were included in the final manuscript
Synthesis approach	Narrative synthesis with explicit distinction between direct PA evidence, German contextual evidence, and indirect conceptual evidence from task-shifting, interprofessional collaboration, and patient-safety literature

Scope definitions and transferability of evidence

The terminology used in this manuscript requires operational clarification. Ambulatory care is the broadest term and refers to physician-led outpatient care delivered outside of inpatient hospital admission. Primary care refers to first-contact, longitudinal, and comprehensive care, typically provided in general practice or family medicine. Specialist outpatient care refers to ambulatory consultations within a defined medical specialty, such as dermatology, cardiology, orthopedics, surgery, or ophthalmology. Post-procedural or postoperative review refers to follow-up care after a known intervention. Stable chronic disease follow-up refers to the structured review of established conditions according to predefined protocols. Undifferentiated first-contact presentations refer to new, incompletely characterized symptoms for which the diagnosis, urgency, and level of clinical risk have not yet been determined.

This distinction is important because the direct empirical evidence supporting the use of PAs is strongest for selected, supervised, and generally less complex primary care or team-based workflows, whereas the proposed German framework applies across a broader ambulatory care setting. Accordingly, the recommendations are strongest for structured, protocol-based, follow-up, or preparation-oriented tasks. By contrast, the recommendations are intentionally more restrictive for undifferentiated first-contact presentations associated with diagnostic uncertainty. In these situations, PA involvement may appropriately include history-taking, measurement of vital signs, clinical documentation, and preparation for physician assessment; however, overall diagnostic and therapeutic responsibility should remain with the supervising physician.

International evidence on PAs in primary and ambulatory care

The international literature suggests that PAs contribute meaningfully to primary and ambulatory care; however, the strength and transferability of the available evidence remain variable. A systematic review by Halter et al. found evidence that PAs can support primary care delivery, although the included studies varied considerably in design, setting, and outcome measures [7]. The mixed-methods study by Drennan et al. remains one of the principal empirical investigations of PA integration into English primary care [8]. It included 12 general practices, 2,086 consultation records, patient surveys, interviews, an economic evaluation, and a video-consultation component. However, the number of PAs included in the primary care component was small, and the data were collected between 2011 and 2012 [8]. These factors limit the direct applicability of the findings to contemporary German ambulatory care, where workload, appointment demand, patient expectations, and regulatory frameworks differ.

A comparison of Physician Associates and general practitioners (GPs) in English primary care reported broadly comparable outcomes in selected same-day consultation settings, although the findings should be interpreted with caution. The study focused on same-day or urgent appointments and involved practices with previous experience employing PAs [9]. Re-consultation rates did not indicate a clear disadvantage for PA consultations; however, differences in case mix and the non-randomized study design limit causal interpretation. Consequently, the findings support the contribution of supervised PAs within selected same-day care pathways but should not be interpreted as evidence that PAs can replace physicians across the full spectrum of undifferentiated primary care presentations.

The video-based comparison of PA and GP consultations provides additional insight into consultation quality and patient safety. In that study, 62 recorded consultations were evaluated after editing by blind assessors to the clinician group whenever possible. Although all consultations were judged to be safe, GPs generally achieved higher ratings on measures of consultation quality [10]. This finding has important implications for German implementation: PA consultations may be safe and clinically appropriate in selected contexts, but physician-level diagnostic breadth and consultation quality should not be assumed to be equivalent across all clinical situations.

Qualitative studies further demonstrate that successful PA integration is both a social and an organizational process. Research on professional boundaries has shown that the PA role may challenge established professional hierarchies and expectations unless responsibilities are clearly negotiated and communicated within the healthcare team [11]. Studies exploring patient experience have reported that patients value access, continuity, and the interpersonal quality of PA consultations, while also showing that many patients do not fully understand the PA role [12]. Jackson et al. conducted interviews and focus groups involving 51 stakeholders, including GPs, advanced nurse practitioners, and patients. GPs expressed concerns regarding case complexity, supervision requirements, prescribing limitations, and medicolegal responsibility, whereas patients were generally accepting of PA involvement when effective physician supervision was evident [13]. Similarly, a service evaluation of PA interns in primary care demonstrated overall acceptability but also identified role ambiguity, a gap between expectations and preparedness, and the need for substantial supervision during the early stages of implementation [14].

Older literature from the United States provides valuable background but should be interpreted cautiously in the German context. Hooker and Everett described the contribution of PAs to primary care, particularly with respect to improving access, expanding service capacity, and supporting team-based care [15]. Economic reviews have suggested that PA models may reduce labor or resource costs in certain settings; however, these findings depend heavily on assumptions regarding substitution, reimbursement systems, scope of practice, and the specific tasks delegated to PAs [16]. In Primary Care Plus models, PAs and nurse practitioners have been evaluated as part of initiatives to shift selected specialist services closer to the community, although the evidence remains heterogeneous and highly context dependent [17]. Broader reviews of PA and advanced practice provider roles within the NHS and hospital-based teams likewise emphasize that successful implementation depends on role design, recruitment, integration, retention, and governance rather than on the introduction of a professional title alone [18-20].

Evidence from team-based care in the United States also provides useful, albeit indirect, insights. Morgan et al. reported that physicians, nurse practitioners, and PAs may influence healthcare utilization and costs differently when caring for patients with complex conditions, underscoring that workforce composition is not a neutral variable and that outcomes depend on patient complexity and team design [21]. Everett et al. found that nurse practitioners and PAs could perform effective roles within multidisciplinary teams caring for Medicare beneficiaries with diabetes; however, these findings are best interpreted as supporting structured team-based contributions rather than unrestricted autonomous substitution in undifferentiated clinical care [22]. Taken together, the international literature supports the feasibility and potential value of integrating PAs into ambulatory care but consistently identifies the same determinants of successful implementation: appropriate patient selection, clearly defined tasks, effective supervision, a well-defined scope of practice, robust documentation, and acceptance by the healthcare team.

Recent UK debate and critical synthesis for Germany

The recent UK debate is particularly relevant because it highlights both the potential benefits and the challenges associated with PA implementation. A 2025 rapid systematic review published in The BMJ emphasized that policy discussions have been complicated by uncertainty regarding the problems that Physician Associates and Anesthesia Associates are intended to address and the roles they should perform [23]. The review identified a limited and heterogeneous evidence base, noted that no studies had directly evaluated safety incidents, and cautioned that the absence of reported safety incidents should not be interpreted as evidence of safety. It also identified primary care as a particularly challenging setting because PAs may practice with greater autonomy, encounter more diverse case mixes, face greater diagnostic uncertainty, have less institutional support, and require more complex supervision than in other clinical environments [23].

A 2025 systematic review focusing specifically on UK primary care provides a more setting-specific synthesis. Willacy et al. searched multiple databases for UK primary care studies published between 2011 and 2024 and included 16 studies [24]. Thirteen studies examined workload, 8 assessed safety, 13 evaluated efficacy, and 8 addressed cost-effectiveness [24]. The review concluded that the published evidence remains limited. Although safety findings were generally reassuring when consultations were assessed objectively, the effects of PAs on workload, efficacy, and cost-effectiveness were less certain. Evidence regarding cost-effectiveness was particularly limited because supervision time, prescription authorization, and indirect workflow costs were often not fully accounted for [24].

A separate rapid review of the quality of care similarly suggested that many studies reported safe and effective PA practice, particularly in settings involving direct supervision and post-diagnostic care. However, the authors cautioned that these findings should not be interpreted as evidence supporting safe autonomous substitution across all clinical settings [25]. The apparent differences between these reviews are informative rather than contradictory. They reflect variations in research questions, inclusion criteria, clinical settings, and standards used to evaluate safety, quality, and value. A cautious synthesis is therefore warranted: PAs can contribute safely and effectively in selected supervised settings, but the current evidence does not support broad claims of automatic workload reduction, cost-effectiveness, or autonomous management of undifferentiated first-contact presentations.

Public understanding of the PA role represents an important patient safety consideration. A systematic review of public perceptions in the United Kingdom found that many patients had a limited understanding of the Physician Associate profession, including confusion between PAs, physicians, and other healthcare professionals [26]. A health policy case study of the UK experience likewise emphasized the importance of careful planning, clearly defined objectives, and consideration of both the benefits and potential challenges when introducing new professional groups into the medical workforce [27]. These findings are particularly relevant to Germany, where public familiarity with the PA role remains limited and where ambulatory care frequently involves undifferentiated, time-sensitive, and diagnostically uncertain presentations.

The 2025 Leng Review provided a policy-level response to these concerns by addressing safety, effectiveness, supervision, role definition, and patient transparency in relation to Physician Associates and Anesthesia Associates in the United Kingdom [28]. The General Medical Council now regulates doctors, Physician Associates, and Anesthesia Associates as distinct professional groups, further reinforcing the importance of maintaining clear professional boundaries rather than allowing role blurring [29]. NHS England has also published its response to the recommendations of the independent review, demonstrating that issues of role clarity, supervision, and safe deployment remain active policy priorities rather than purely academic concerns [30]. For Germany, the most transferable lesson is not the UK regulatory model itself but the underlying safety principle: the greater the ambiguity surrounding a professional role, the greater the need for explicit governance.

Germany-specific context

German evidence on Physician Assistants (PAs) remains limited compared with that available from the United States and the United Kingdom. A cross-sectional survey of PA employment and workforce entry in Germany described the development of the profession and provided early evidence on employment patterns, educational pathways, and job satisfaction [31]. Meyer-Treschan et al. conducted one of the few empirical German studies examining collaboration between PAs and physicians. The study included seven PAs and seven delegating physicians from a single PA graduate cohort, together with a retrospective analysis of process times in an interdisciplinary emergency department [32]. Physicians who had worked with PAs for several years reported reduced workload and high levels of satisfaction, while PAs described a broad range of clinical responsibilities and high professional satisfaction. In the emergency department analysis, waiting times and overall length of stay were not prolonged and were shorter when a PA was involved. However, the study was small, conducted predominantly in a hospital setting, and was not designed to generate generalizable evidence for ambulatory care [32].

A separate German publication distinguished the PA profession from physicians in postgraduate training and provides important legal and conceptual clarification [33]. The authors emphasized that PAs may perform only delegated medical tasks, are not licensed to practice medicine independently, and do not progress toward independent specialist responsibility in the same manner as physicians undergoing postgraduate specialty training. The publication also distinguished preparatory and supportive activities from responsibilities reserved exclusively for physicians. PAs may perform preparatory history-taking, preliminary clinical examinations, supportive patient counseling, and assistance with invasive therapies or procedures. However, they should not independently determine indications for treatment, establish diagnoses, obtain legally valid informed consent, make treatment decisions, or perform physician-reserved core clinical activities [33]. This distinction directly supports the delegation framework proposed in the present manuscript.

Treusch et al. reported that German PAs undertake a broad range of clinical activities, including documentation, history-taking, and diagnostic services, while also identifying differences in perceived empowerment, career development opportunities, and job-related well-being across practice settings [34]. These findings are important because they demonstrate that the PA role may be experienced differently across specialties and healthcare environments. They also suggest that supervision quality, team dynamics, and opportunities for professional development are not peripheral considerations but may influence workforce retention, professional satisfaction, and, ultimately, continuity of patient care.

The German ambulatory care sector is highly heterogeneous. Rural general practices, specialist dermatology practices, cardiology group practices, and multi-site medical care centers differ substantially in case mix, diagnostic uncertainty, staffing, supervision capacity, procedural workload, and patient expectations. Consequently, a single generic PA model is unlikely to be appropriate across all settings. Instead, PA integration should be tailored to the specialty, practice environment, and demonstrated competencies of the individual PA.

The position paper of the German Medical Association is particularly important because it places PAs firmly within a physician-led delegation model while explicitly preserving the distinction between delegable activities and responsibilities reserved for physicians [5]. The delegation framework of the Federal Association of Statutory Health Insurance Physicians is equally important because it formalizes the requirements for delegating medical services to qualified non-physician personnel in statutory ambulatory care [4]. Together, these sources support a model in which the PA functions neither as an autonomous medical practitioner nor as a purely administrative assistant. Rather, the PA is a qualified member of the physician-led healthcare team whose clinical contribution depends on clearly defined delegation, assessment of individual competence, and appropriate physician supervision.

German experience with other non-physician roles in ambulatory care also provides a useful point of comparison. A cross-sectional study of task delegation within German general practice teams found that GPs were willing to delegate selected responsibilities to specially qualified practice staff, whereas more complex clinical assessments remained physician responsibilities [35]. Similarly, a regional survey of German GPs reported willingness to delegate home visits to appropriately trained assistants but also identified financial considerations, training requirements, and informal delegation practices as important barriers and facilitators to implementation [36]. Although these studies do not directly validate the PA model, they demonstrate that Germany already has practical experience with supervised delegation and that physician attitudes, education, reimbursement, and clarity of professional responsibility are key determinants of successful implementation.

The Act on the Expansion of Competencies and Reduction of Bureaucracy in Nursing [6] likewise reflects a broader policy interest in expanding the roles of non-physician health professionals in Germany. However, because this legislation primarily addresses the nursing profession, it does not establish a regulatory framework for PAs. The PA role should therefore not be conflated with nursing, advanced nursing practice, or nursing competency reforms. Instead, it requires a distinct framework for supervision and delegation that remains firmly anchored in physician-led responsibility.

Table 2 summarizes the directness, limitations, and practical implications of the main evidence domains informing German ambulatory implementation.

**Table 2:** Evidence strength and transferability for German ambulatory implementation Source: Author synthesis based on direct physician assistant (PA) literature, German professional and regulatory sources, UK policy reviews, German delegation studies, and indirect patient-safety and task-shifting literature [4-42].

Evidence area	Directness for German ambulatory care	Main contribution	Key limitations	Practical implication
German legal and professional sources [4-6]	Direct contextual evidence	Defines delegation logic, physician responsibility, and PA role boundaries in Germany	Policy and professional documents are not outcome studies	Use as the regulatory and governance foundation, not as proof of effectiveness
English primary-care PA studies [8-14,24]	Direct for UK primary care; indirect for Germany	Shows the feasibility of PA work in selected same-day, routine, and supervised primary-care workflows; highlights patient acceptability and integration barriers	Small numbers of PAs, older data in several studies, non-randomized designs, selected practices, different UK regulations and workforce context	Transfer cautiously; begin with selected patient groups, onboarding, supervision time, and explicit escalation
UK safety debate, public perception, and policy review [23,26-30]	Indirect but highly relevant safety context	Identifies role ambiguity, patient confusion, supervision concerns, and uncertainty about the scope of practice as safety and trust issues	UK controversy and regulation are not identical to Germany	Make patient-facing role clarity and named physician supervision mandatory components
German PA empirical literature [31-34]	Direct German evidence, but limited ambulatory outcome evidence	Shows early employment patterns, physician satisfaction, PA activity profiles, and conceptual differentiation from physicians in training	Small samples, limited ambulatory data, often hospital-based or descriptive	Use to define the German role and gaps; avoid strong claims about ambulatory outcomes
German delegation studies involving other non-physician roles [35,36]	Indirect German ambulatory evidence	Shows that task delegation can be accepted in German general practice when training, costs, and responsibility are addressed	Not PA-specific and often focused on other assistant roles	Use as implementation analogy only
Task-shifting, interprofessional collaboration, diagnostic safety, and handover literature [37-42]	Indirect conceptual evidence	Supports principles of task definition, supervision, escalation, structured communication, and diagnostic safety	Does not directly measure PA outcomes in German ambulatory care	Use to support safety architecture, not as direct evidence of PA effectiveness

Delegation versus substitution

The distinction between delegation and substitution is fundamental to the safe integration of PAs. Delegation refers to a physician assigning defined clinical tasks to a qualified individual while retaining overall clinical responsibility. Substitution, by contrast, implies the replacement of physician-level clinical decision-making. Within German ambulatory care, the defensible model is one of delegated participation in physician-led care rather than the autonomous replacement of physicians in the assessment of undifferentiated clinical presentations.

This distinction is not merely semantic. Ambulatory presentations frequently begin with incomplete information, nonspecific symptoms, and uncertain levels of clinical risk. Symptoms such as chest pain, dizziness, abdominal pain, dyspnea, fatigue, headache, skin lesions, and psychiatric complaints may represent either benign conditions or serious underlying disease. The ability to recognize red flags, manage diagnostic uncertainty, formulate appropriate differential diagnoses, and determine when protocol-based care is insufficient constitutes a core component of medical training and clinical practice. PAs can make valuable contributions to structured patient assessment, history-taking, physical examination, documentation, follow-up care, preventive services, and chronic disease management. However, the boundary between delegated clinical support and independent diagnostic responsibility must remain explicit.

The broader literature on task shifting in primary care reinforces this principle but should not be overinterpreted. A Cochrane review evaluating nurses as substitutes for physicians found that appropriately trained nurses could achieve outcomes comparable to, or, in some cases, better than those of physicians for selected primary care tasks. However, the effects on costs and several process-related outcomes remained uncertain and were highly dependent on role design and healthcare context [37]. Similarly, a systematic review and meta-analysis of physician-nurse substitution concluded that such models may be clinically effective in selected primary care settings, but the findings should not be interpreted as supporting the wholesale replacement of physicians. Rather, they demonstrate that carefully selected clinical tasks can be redistributed safely under appropriate qualifications, supervision, and healthcare system conditions [38]. As these studies evaluated nursing rather than PA practice, they provide only indirect conceptual support for the framework proposed in the present manuscript.

A delegation model can reduce physician workload only if supervision is designed realistically. Supervision itself requires time and organizational resources. Hidden supervisory demands, frequent informal interruptions, unclear allocation of responsibility, and the retrospective correction of clinical decisions may ultimately increase, rather than reduce, physician workload. Consequently, the PA role should be integrated into clinical workflows that clearly define which patient groups are appropriate for PA-supported care, when physician review is mandatory, and how documentation, communication, and follow-up responsibilities are managed.

Patient safety considerations

Patient safety risks may arise when professional role boundaries are unclear or when PAs are expected to practice beyond the limits of their training or supervision. The most relevant risks include diagnostic delay, failure to recognize red flags, inadequate escalation, patient misunderstanding of professional roles, deficiencies in clinical documentation, overreliance on protocol-based care in non-standard clinical presentations, and hidden physician workload resulting from supervision that is not formally recognized or appropriately accounted for.

Diagnostic risk warrants particular attention in ambulatory care. Outpatient diagnostic error has been estimated to affect a substantial number of patients in US primary care and is recognized as a major patient safety concern [39]. Although these findings cannot be directly extrapolated to PA-supported ambulatory care in Germany, they underscore that undifferentiated outpatient presentations should not be regarded as inherently low risk simply because they occur outside the hospital setting. Accordingly, any PA model implemented in German ambulatory care should incorporate clearly defined escalation criteria and explicit triggers for physician review.

Interprofessional collaboration has the potential to improve clinical practice and patient outcomes, although systematic reviews indicate that the evidence base is complex and that the effectiveness of individual interventions varies according to context [40]. Structured communication tools such as SBAR can facilitate escalation and promote shared situational awareness among healthcare professionals [41]. Evidence on standardized clinical handover likewise supports the broader principle that structured communication can reduce information omissions and strengthen patient safety processes [42]. These findings are transferable to PA integration only as indirect patient safety principles. They do not constitute evidence of PA effectiveness but reinforce the importance of standardized escalation pathways and documentation practices when delegated clinical responsibilities are incorporated into routine care.

Overall, the synthesis of the available literature supports a cautious approach. PA-supported care is most defensible when integrated within a physician-led model characterized by clearly defined task boundaries, direct access to physician supervision, transparent communication with patients regarding professional roles, and ongoing quality monitoring. By contrast, the current evidence provides less support for broad claims that PAs automatically reduce physician workload, improve cost-effectiveness, or safely manage undifferentiated first-contact presentations without structured supervision. The framework proposed in this manuscript is intended to translate this cautious interpretation of the evidence into a practical model for implementation.

A practical supervision framework for German ambulatory care

This review proposes a six-domain framework for the safe integration of PAs into German ambulatory care: role transparency, a delegation matrix, tiered supervision, escalation criteria, documentation standards, and quality monitoring. The framework is intended as an evidence-informed starting point for implementation in general practices, specialist practices, professional practice partnerships, and medical care centers. It should be adapted according to the clinical specialty, practice size, physician availability, PA experience, and applicable legal requirements.

Role transparency requires that patients clearly understand whether they are being seen by a physician or a PA. This distinction should be communicated consistently through appointment booking systems, waiting room information, name badges, verbal introductions, and clinical documentation. A standardized introduction may be: "I am a Physician Assistant working as part of this physician-led medical team. I will take your history and perform your examination, and I will discuss the next steps with the responsible physician." This wording avoids both minimizing and overstating the PA role.

A delegation matrix should classify clinical tasks into four categories: generally delegable, delegable following individual competence assessment, delegable only with direct physician involvement, and not appropriate for autonomous PA management. The matrix should be documented in writing, tailored to the individual practice, and reviewed regularly. It should take into account task complexity, clinical risk, patient vulnerability, PA experience, and the availability of physician supervision.

Supervision terminology should be operationally defined. Direct observation refers to the physician observing the PA-patient interaction or being physically present during the relevant portion of the encounter. Direct physician assessment refers to the physician personally reassessing the patient before making the final diagnostic or therapeutic decision. Same-day case discussion refers to the PA presenting the case to the supervising physician before the patient leaves the practice or before any final clinical decision is implemented. Record-based confirmation refers to a physician review of the PA's documentation and management plan, typically for low-risk, protocol-based cases. Indirect supervision means that the physician is available for consultation but does not routinely review every low-risk encounter. Escalation-triggered review requires mandatory physician involvement whenever predefined red flags, diagnostic uncertainty, abnormal findings, or a patient request for physician review is present.

Escalation criteria should be explicit, standardized, and documented. Immediate physician assessment should be required for patients presenting with red-flag symptoms, diagnostic uncertainty, abnormal vital signs, new severe pain, neurological deficits, chest pain, dyspnea, syncope, suspected sepsis, acute psychiatric risk, unexplained clinical deterioration, vulnerable patient characteristics, complex multimorbidity, or any situation in which the PA is uncertain about the appropriate course of action.

Documentation standards should clearly identify the PA as the healthcare professional responsible for the clinical record and indicate when the case was discussed with or reviewed by the supervising physician. Physician review should be documented whenever cases involve diagnostic uncertainty, treatment initiation outside predefined protocols, abnormal clinical findings, escalation criteria, or a patient request for physician involvement.

Quality monitoring should be practical, proportionate, and integrated into routine clinical governance. Relevant indicators include the number and type of PA-supported patient encounters, escalation frequency, physician review rates, documentation completeness, patient complaints related to role confusion, re-consultation following PA-supported care, adverse events, patient satisfaction, and physician-reported supervision workload. These indicators should be reviewed regularly so that the PA role can be refined proactively before potential safety concerns become embedded in routine practice.

Table 3 summarizes the proposed operational delegation and supervision matrix for German ambulatory care, including definitions, appropriate settings, required physician actions, and documentation examples.

**Table 3:** Operational delegation and supervision matrix for German ambulatory care Source: Author synthesis based on German delegation requirements, the German Medical Association position paper, German physician assistant (PA) literature, UK primary-care and policy literature, diagnostic-safety literature, and structured communication or handover principles [4,5,8-14,23-30,32-42].

Level or term	Operational definition	Appropriate tasks or settings	Physician action required	Documentation example
Generally delegable after onboarding	Task can be performed by a PA after local induction and written task instruction; physician remains available for questions	Structured history-taking, vital signs, medication reconciliation, preventive-care preparation, documentation support, patient education using approved materials, referral coordination	Physician availability during the session; periodic review according to local protocol	PA assessment documented; no red flags; physician available
Delegable after individual competence assessment	Task requires documented competence beyond onboarding and should be linked to protocols or recurrent workflows	Stable chronic-disease follow-up, wound checks, post-procedural review, preparation of diagnostic workups, structured review of known conditions	Scheduled periodic case review; immediate escalation if criteria are met	PA review under protocol; competence documented; no escalation criteria met
Direct observation	Physician observes the PA-patient interaction or the relevant examination/procedure component	Early onboarding, new task acquisition, procedural skills within legal and training limits, high-learning-value encounters	Physician physically present or directly observing the relevant component	Observed by supervising physician; feedback given
Same-day case discussion	PA discusses the case with the supervising physician before the patient leaves or before the final clinical action is taken	Moderate-risk follow-up, non-standard findings, new but apparently low-risk symptoms, uncertainty about next steps	Physician discusses case contemporaneously and confirms or modifies the plan	Discussed with Dr X same day; plan agreed
Direct physician assessment	Physician personally reassesses the patient before final diagnosis, treatment decision, referral decision, or discharge from the encounter	Red flags, abnormal vital signs, diagnostic uncertainty, complex multimorbidity, vulnerable patients, new severe symptoms, deterioration	Physician sees and assesses the patient personally	Physician examined patient; final diagnosis and plan by physician
Record-based confirmation	Physician reviews documentation and plan without necessarily reassessing the patient, appropriate only for low-risk structured tasks	Protocol-based follow-up with normal findings, documentation quality checks, stable known condition without escalation triggers	Physician reviews record according to protocol or audit schedule	Record reviewed by physician; no further action required
Indirect supervision	Physician is available for consultation but does not routinely review every low-risk encounter	Experienced PA, low-risk protocol-based follow-up, administrative-clinical tasks, patient education, care coordination	Physician available during session or by defined backup route	Indirect supervision; escalation criteria not met
Not suitable for autonomous PA management	Task should not be finalized by a PA without physician decision-making because it involves physician-reserved or high-risk clinical responsibility	Final diagnosis and treatment responsibility for undifferentiated high-risk symptoms, independent red-flag management, unsupervised prescribing decisions, legally physician-reserved decisions, invasive procedures beyond training and authorization	Physician assessment and final decision-making required; PA may assist with preparation, documentation, and follow-up	PA prepared case; physician made final diagnostic and therapeutic decision

Implementation checklist for German ambulatory settings

Before integrating a PA into a German ambulatory care setting, a practice or medical care center should consider the following operational questions. Is the PA role clearly explained to patients before and during the clinical encounter? Is there a written, specialty-specific delegation matrix? Is a named supervising physician assigned for every PA session? Are supervision levels defined according to task complexity, PA experience, and patient risk? Are red-flag symptoms and escalation criteria documented and readily accessible within routine clinical workflows? Is PA documentation clearly distinguishable from physician documentation? Is there a process for same-day physician case discussion or direct physician assessment when required? Can patients request physician review without unnecessary barriers? Are patient safety, workflow, and patient understanding monitored using predefined indicators? Is the PA role reviewed periodically and refined on the basis of clinical outcomes, supervision experience, and stakeholder feedback?

This checklist is intentionally practical. The successful implementation of PAs cannot rely solely on workforce planning or strategic policy initiatives. Instead, it must be translated into everyday clinical practice through appointment scheduling, triage protocols, documentation templates, protected supervision time, patient information, staff training, and ongoing quality review. Practices should initially limit PA responsibilities to low-risk, structured clinical tasks and expand the scope of delegated activities only after competence, reliable supervision, and patient understanding have been clearly demonstrated.

Limitations

This article is a narrative review and was not intended to provide a systematic evidence synthesis or meta-analysis. Although the search strategy was described in greater detail in response to peer review, the review remains selective and interpretive by design. The literature on PAs is heterogeneous, and findings from one healthcare system cannot be directly extrapolated to another. Definitions, educational pathways, supervision models, prescribing authority, regulatory frameworks, reimbursement structures, and public understanding differ substantially across countries.

The inclusion of literature on task shifting and physician-nurse substitution provides conceptual support for the principles of delegation and supervision but should not be interpreted as direct evidence of PA effectiveness or clinical outcomes. Likewise, evidence relating to SBAR, clinical handover, and interprofessional collaboration supports the principles of structured communication and escalation but does not validate the proposed supervision framework for PAs in German ambulatory care.

German evidence on PA-supported ambulatory care remains limited. Consequently, the proposed framework should be regarded as an evidence-informed, practice-oriented starting point rather than as a validated intervention. Future research should evaluate the framework prospectively in German ambulatory care settings using outcomes such as patient safety, diagnostic quality, physician workload, supervision burden, patient understanding of professional roles, staff satisfaction, service capacity, and cost-effectiveness. In addition, the framework should undergo legal and contractual review before local implementation, as delegation requirements may vary according to practice setting, medical specialty, payer environment, and future regulatory developments.

## Conclusions

PAs may become a useful component of German ambulatory care, but only if their integration is based on structured delegation, clear supervision, patient transparency, and physician-led governance. International experience suggests that role ambiguity and inadequate supervision are central safety risks. A German implementation model should therefore avoid framing PAs as autonomous physician substitutes in undifferentiated ambulatory care. Instead, PAs should be integrated as supportive members of physician-led teams, with defined task boundaries, explicit escalation criteria, and continuous quality monitoring.

The proposed supervision framework offers an evidence-informed starting structure for general practices, specialist practices, professional practice partnerships, and medical care centers. Its central message is deliberately conservative: the value of PAs depends not on maximal autonomy, but on safe role design. Clear delegation, transparent communication, reliable escalation, and documented physician responsibility may support safer implementation and more coherent team workflows. However, the effects of this model on capacity, workload, cost-effectiveness, patient safety, patient understanding, and continuity of care require pilot implementation and prospective evaluation before stronger claims can be made.
